# FGF21 alleviates neuroinflammation following ischemic stroke by modulating the temporal and spatial dynamics of microglia/macrophages

**DOI:** 10.1186/s12974-020-01921-2

**Published:** 2020-08-31

**Authors:** Dongxue Wang, Fei Liu, Liyun Zhu, Ping Lin, Fanyi Han, Xue Wang, Xianxi Tan, Li Lin, Ye Xiong

**Affiliations:** 1 Department of Neurosurgery, First Affiliated Hospital of Wenzhou Medical University, Wenzhou, 325035 Zhejiang China; 2 School of Pharmaceutical Sciences, Wenzhou Medical University, Wenzhou, 325035 Zhejiang China

**Keywords:** rhFGF21, Stroke, Neuroinflammation, Microglia/macrophage, NF-κB, PPAR-γ

## Abstract

**Background:**

Resident microglia and macrophages are the predominant contributors to neuroinflammation and immune reactions, which play a critical role in the pathogenesis of ischemic brain injury. Controlling inflammatory responses is considered a promising therapeutic approach for stroke. Recombinant human fibroblast growth factor 21 (rhFGF21) presents anti-inflammatory properties by modulating microglia and macrophages; however, our knowledge of the inflammatory modulation of rhFGF21 in focal cerebral ischemia is lacking. Therefore, we investigated whether rhFGF21 improves ischemic outcomes in experimental stroke by targeting microglia and macrophages.

**Methods:**

C57BL/6 mice were subjected to middle cerebral artery occlusion (MCAO) and randomly divided into groups that received intraperitoneal rhFGF21 or vehicle daily starting at 6 h after reperfusion. Behavior assessments were monitored for 14 days after MCAO, and the gene expression levels of inflammatory cytokines were analyzed via qRT-PCR. The phenotypic variation of microglia/macrophages and the presence of infiltrated immune cells were examined by flow cytometry and immunostaining. Additionally, magnetic cell sorting (MACS) in combination with fluorescence-activated cell sorting (FACS) was used to purify microglia and macrophages.

**Results:**

rhFGF21 administration ameliorated neurological deficits in behavioral tests by regulating the secretion of pro-inflammatory and anti-inflammatory cytokines. rhFGF21 also attenuated the polarization of microglia/macrophages toward the M1 phenotype and the accumulation of peripheral immune cells after stroke, accompanied by a temporal evolution of the phenotype of microglia/macrophages and infiltration of peripheral immune cells. Furthermore, rhFGF21 treatment inhibited M1 polarization of microglia and pro-inflammatory cytokine expression through its actions on FGF receptor 1 (FGFR1) by suppressing nuclear factor-kappa B (NF-κB) and upregulating peroxisome proliferator-activated receptor-γ (PPAR-γ).

**Conclusions:**

rhFGF21 treatment promoted functional recovery in experimental stroke by modulating microglia/macrophage-mediated neuroinflammation via the NF-κB and PPAR-γ signaling pathways, making it a potential anti-inflammatory agent for stroke treatment.

## Introduction

Ischemic stroke, reduced cerebral blood flow caused by an arterial thrombus, which afflicts approximately 795, 000 individuals worldwide each year, and the number of patients suffering stroke is rising [1, 2]. However, the therapeutic options for stroke are desperately limited. Slow and incomplete recovery is compounded by limited drug treatments that facilitate the recovery process. Acute care mainly depends on thrombolytic treatment by administrating tissue plasminogen activator (tPA), although the narrow therapeutic time window of within 6 h ensures that only a small fraction of patients benefit [3]. Furthermore, reperfusion of the ischemic brain is considered a secondary injury, and the efficacy of tPA treatment is inconsistent among individuals. Consequently, novel and effective drug treatments that improve the symptoms and sequelae of stroke, especially in acute phases, are urgently needed [4].

Inflammation is a critical component of the secondary injury resolution process under ischemic brain insult. In the event of stroke, microglia residing in the central nervous system (CNS) are the first responders to cerebral ischemia, and they are activated within several minutes [5, 6]. Subsequently, infiltrating immune cells, including monocytes, macrophages, neutrophils, lymphocytes, and natural killer (NK) cells, pass through the disrupted blood-brain barrier (BBB) and secrete a plethora of cytokines to promote the progression of inflammation [7, 8]. Therefore, understanding the contribution of those immune cells to the immunomodulation reaction is a prerequisite for therapeutic intervention. In particular, resident microglia, as well as invading macrophages, are commonly recognized as vital contributors to inflammatory circumstances under the pathophysiology of ischemic stroke [9]. Morphological transformation and common antigens expressed on both cell types give them certain overlapping functions, such as phagocytosis and analogous polarization abilities toward M1- or M2-like phenotypes [6, 10]. The diverse phenotypes distinctively impact the expression of inflammatory cytokines, which are correlated with neuronal functions. Generally, the M1 microglia/macrophages, marked CD16/32 and CD68, are commonly characterized by pro-inflammatory effects accompanied by the release of pro-inflammatory cytokines including tumor necrosis factor-α (TNF-α), inducible nitric oxide synthase (iNOS), interleukin-1β (IL-1β), and interleukin-6 (IL-6), whereas microglia with the M2 phenotype (marked by CD206) secrete transforming growth factor beta (TGF-β), insulin-like growth factor 1 (IGF-1), interleukin (IL)-10, and IL-4 to rescue local inflammation and favor tissue repair [11, 12]. Furthermore, the M2 phenotype is divided into three subsets: M2a (marked by CD206 and arginase-1) is associated with anti-inflammation and immunity against parasites, M2b (marked by CD86 and SOCS3) is related to adaptive immunity, and M2c (marked by TGF-β and IL-10) facilitates tissue regeneration [13]. Notably, the unique temporal and spatial changes in microglia and macrophages under pathophysiological conditions indicate that each cell type has indispensable and complementary roles in the context of ischemic stroke. A growing number of studies have focused on the phenotypic moderation of microglia/macrophages rather than the exclusive suppression of their activation. However, the participation of invading immune cells is usually obscured.

Fibroblast growth factor 21 (FGF21), as a novel and potent regulator of glucose uptake and lipid metabolism, is predominantly expressed in both the rodent and human liver and thymus [14]. Compared with other FGFs, FGF21 scarcely has mitogenic effects and may be the only FGF that can cross the BBB due to its weak binding affinity with heparin [15, 16]. To date, accumulating evidence has indicated that FGF21 exhibited therapeutic effects in multiple disease models, such as atherosclerosis [17], diabetic cardiomyopathy [18], age-related disorders [19], and enhanced neurite outgrowth [20]. Although the mechanisms underlying its pharmacologic actions remain elusive, the therapeutic mechanism of FGF21 primarily involves anti-inflammation [21], energy metabolism and vascular homeostasis [22], oxidative stress [23], and tissue repair [24]. FGF21 mediates these effects by interacting with FGF receptors (mainly FGFR1 and FGFR2) via binding a cofactor, β-klotho, a single-pass transmembrane protein from the klotho family [25]. FGFR1 and its coreceptor β-klotho have also been reported to be widely observed in brain tissue, including microglia [26]. Therefore, FGF21 may have a potential impact on microglia. Additionally, our previous study demonstrated that FGF21 effectively upregulated the downstream effector peroxisome proliferator-activated receptor (PPAR)-γ in human bone marrow endothelial cells [27] and activated PPAR-γ was closely associated with microglial phenotype and inflammatory regulation in the CNS [28]. Moreover, a recent study confirmed that FGF21 suppressed macrophage-mediated inflammation by nuclear factor-erythroid 2-related factor 2 (Nrf2) and the NF-κB signaling pathway in a collagen-induced arthritis model [29].

Therefore, FGF21 is likely to regulate the stroke-induced immune-inflammatory response by modulating microglia and macrophages both in the brain and in peripheral tissue in favor of functional recovery. Recently, recombinant human FGF21 (rhFGF21) has been reported to modulate the shift of microglia from M1 activation to M2 activation at the subacute and chronic stages of db/db mice with middle cerebral artery occlusion (MCAO), which is accompanied by the activation of PPAR-γ in the peri-infarct area [30]. However, the potential mechanism by which FGF21 acts on microglia/macrophages and the dynamic alteration of microglia/macrophages and their phenotypes have not been elucidated. In the current study, we investigated the neuroprotective effect and promising mechanism by which FGF21 ameliorates inflammatory responses and microglia/macrophage polarization in a mouse model of MCAO.

## Materials and methods

### Reagents and antibodies

rhFGF21 was supported by the laboratory of Biotechnology Pharmaceutical Engineering at Wenzhou Medical University and synthesized on the basis of the study previously reported [31]. Antibodies in flow cytometry analysis involving CD3-PE (17A2, 100206), CD3-PerCP-Cy5.5 (17A2, 100217), CD8-FITC (53-5.8, 140404), CD4-APC (GK1.5, 100412), F4/80-FITC (BM8, 123108), NK1.1-APC (PK136, 180710), Ly6G-PE (1A8, 127608), Ly6C-APC (HK1.4, 128016), CD45-APC (103112), CD11b-PE (101208), CD206-APC (C068C2, 141708), CD206-FITC (C068C2, 141704), CD68-PerCP-Cy5.5 (FA-11, 137014), CD86-PE (GL-1, 105008), CD45-PE/Cy7 (30-F11, 103114), CD11b-PE/Cy7 (M1/70, 101216), and CD11b-PerCP-Cy5.5 (M1/70, 101230) were purchased from BD Biosciences (San Jose, CA, USA) and CD45-APC (OX33, 17046280), CD11b-PE (OX42, 12011080), and CD86-FITC (24F, 11086081) were purchased from eBioscience (San Diego, CA, USA).

Antibodies applied in immunofluorescence including CD16/32(AF1460) and CD206 (AF2535) were purchased from R&D Systems (Minneapolis, MN, USA) and Iba1 (019-19741) purchased from Wako pure chemical corporation (Tokyo, Japan).

The primary antibodies applied in western blot including anti-NF-κB (3033T), anti-FGFR1 (ab824), anti-p-FGFR1 (ab59194), anti-PPAR-γ (ab28364), and anti-β-Actin (ab8227) were purchased from Cell Signaling Technology (Danvers, MA, USA) or Abcam (Cambridge, MA, USA). The secondary antibodies used were donkey anti-rabbit IgG H&L (HRP) (ab150075) or goat anti-mouse IgG H&L (HRP) (ab150115), which were commercially purchased from Abcam (Cambridge, MA, USA).

Corresponding reagent or kit applied in this study include trizol reagent (Qiagen, Duesseldorf, Germany), PrimeScript^TM^ RT Reagent Kit (TaKaRa, Shiga, Japan), iQ^TM^ SYBR Green supermix (Bio-Rad, Hercules, CA, USA), miRNeasy Micro Kit (Qiagen, Duesseldorf, Germany), QuantiTect Reverse Transcription kit (Qiagen, Duesseldorf, Germany), TaqMan® Gene Expression Assays (ThermoFisher Scientific, Fremont, CA, USA), Neural Tissue Dissociation Kits (Miltenyi Biotech, Bergisch Gladbach Germany), and Fluoroshield mounting medium with DIPI (Abcam, Cambridge, MA, USA).

### Animal groups and drug administration

C57BL/6 mice (20–25 g) were purchased from the Animal Center of the Chinese Academy of Science (Beijing, China), and all surgical procedures and experimental protocols were approved by the Animal Care and Use Committee of Wenzhou Medical University. All animals were randomly assigned to the following three groups by a randomized block design: sham group, MCAO group, and MCAO+rhFGF21 group. In the sham group, mice were subjected to the same anesthesia and surgical procedures as the other groups but the filament was not inserted. In the MCAO+rhFGF21 group, the mice were intraperitoneally injected with rhFGF21 once per day at a dose of 1.5 mg/kg for 7 consecutive days beginning at 6 h after reperfusion.

### Transient focal cerebral ischemia and reperfusion model preparation

The surgical procedures to establish the MCAO model were based on the intraluminal filament technique [32]. Briefly, the mice were anesthetized by isoflurane and placed on a heating blanket to maintain body temperature at 37 ± 0.5 °C. A midline incision was made to expose the common carotid artery (CCA), external carotid artery (ECA), and internal carotid artery (ICA). The CCA was temporarily closed and a monofilament (0.18 ± 0.01 mm, Jialing Biotechnology Company, Guangdong, China) was inserted into the ICA through the ECA until it reached the middle cerebral artery, and it was left for 60 min. Laser Doppler flowmetry (model P10, Moor Instruments, Wilmington, DE, USA) was used to monitor whether cerebral flow dropped to lower than 20% of the pre-ischemic level. The occluding filament was returned to the ICA to achieve reperfusion after 60 min of occlusion. In the MCAO model, the mortality rate was 9.3% (23 of total 246) and exclusion rate was 10.9% (11 of the total 246 experienced inadequate reperfusion, and 16 of the total 246 reached the criteria limitations set for the modified Neurological Severity Score (mNSS) scoring system, i.e., mNSS scores < 6 or > 13 at 24 h after MCAO were excluded).

### Neurological function assessment

The mNSS, rotarod test, corner-turning test, and adhesive removal test were performed to assess neurodeficits, motor coordination, sensorimotor asymmetry, and feeling functions at 1, 3, 7, and 14 days after surgery. All animals received training for 3 consecutive days before suffering ischemia-reperfusion injury. Behavior data were recorded as preoperative data at the second day after training. Subsequently, a transient focal cerebral ischemia and reperfusion model about MCAO was performed on the following day. The assessment procedure was performed by the same investigator who was blinded to the group identity of each mouse.

### Quantitative real-time PCR

Total mRNA was isolated from the cortex samples around the infarcted zone using trizol reagent according to the manufacturer’s instructions. The cDNA was synthesized by the PrimeScript^TM^ RT Reagent Kit (TaKaRa, Shiga, Japan) following the manufacturer’s protocol. PCR assays were performed on a CFX Connect Real-time System (Bio-Rad, Hercules, CA, USA) using SYBR Green. The primers used in this study are shown in Table 1. Additionally, total RNA was extracted from the sorted microglia using the miRNeasy Micro Kit according to the manufacturer’s protocol. cDNA was transcribed with a Reverse Transcription kit and amplified in step one using Gene Expression Assays for TNF-α, IL-6, IL-1β, and TGF-β. The reaction volume was set to 20 μl and performed at 50 °C for 2 min, 95 °C for 20 s, followed by 40 cycles of 1 s at 95 °C and 20 s at 60 °C. Data were analyzed using the 2^-∆∆Ct^ method, and the expression level of relative mRNA was then reported as the fold difference.

**Table 1:** PPrimers sequences for qRT-PCR

Gene	Primer sequences (5′ to 3′)
β-Actin	Forward CACTGCAAACGGGGAAATGG
	Reverse TGAGATGGACTGTCGGATGG
IL-1β	Forward GCG CTG CTC AAC TTC ATC TTG Reverse GTG ACA CAT TAA GCG GCT TCA C
IL-6	Forward CTC CCA ACA GAC CTG TCT ATA C
	Reverse CCA TTG CAC AAC TCT TTT CTC A
TNF-α	Forward GTG ACA AGC CTG TAG CCC A
	Reverse ACT CGG CAA AGT CGA GAT AG
Cox-2	Forward CCCTTGGGTGTCAAAGGTAA
	Reverse GCCCTCGCTTATGATCTGTC
MCP-1	Forward ATAGCAGCCACCTTCATTCC Reverse TTCCCCAAGTCTCTGTATCT
CXCL1	Forward ACC GAA GTC ATA GCC ACA CCTC AAG Reverse TTG TCA GAA GCC AGC GTT CAC C
IL-10	Forward TTC TTT CAA ACA AAG GAC CAG C
	Reverse GCA ACC CAA GTA ACC CTT AAA G
TGF-β	Forward TTGCTTGAGCTCCACAGAGA
	Reverse TGGTTGTAGAGGGCAAGGAC

### Flow cytometry

After the mice were euthanized, fresh brain, spleen, and blood tissues were harvested for single-cell suspension preparation for subsequent single-cell analysis using fluorochrome-conjugated antibodies. Spleen and blood tissues were dissociated into single-cell suspensions as previously described [33, 34]. Splenocytes were dissociated by sieving through a 70-μm filter, and then lysing solution (BD Bioscience, CA, USA) was used to deplete red blood cells in the spleen and blood. Brain mononuclear cells were prepared by Neural Tissue Dissociation Kits (Miltenyi Biotech, Bergisch Gladbach Germany) according to its protocol. Briefly, the ischemic hemisphere of the brain was collected and dissected into small pieces. The pieces were pipetted back into an appropriate-sized conical tube, rinsed with cold Hank’s balanced salt solution (HBSS), and then centrifuged (300 *g*, 2 min) at room temperature. After the supernatant was carefully aspirated, preheated enzyme mix1 (37 °C, 10 min) in a Neural Tissue Dissociation Kit was added to digest tissue pieces for 15 min, and then preheated enzyme mix 2 (37 °C, 10 min) was added to the tissue sample for 10 min. Subsequently, HBSS was used and single pellets were isolated by passing through a 30-μm cell strainer. Cell pellets obtained from the spleen, blood, and brain were washed and incubated with antibodies targeting CD3, CD8, CD4, F4/80, NK1.1, Ly6G, Ly6C, CD45, CD11b, CD206, CD68, and CD86, and tagged with phycoerythrin (PE), fluorescein isothiocyanate (FTIC), allophycocyanin (APC), PerCP-Cy5.5, or PE-Cy7. Antibody staining was performed following the manufacturer’s protocol. Fluorescence-minus-one (FMO) controls were used to determine the gate of each antibody. Flow cytometry analysis was conducted using a FACS Aria flow cytometer (BD Bioscience, CA, USA), and data were analyzed by FlowJo software (Informer Technologies, USA).

### Sorting of microglia and macrophages

Microglia of the mouse brain tissue were sorted by magnetic cell sorting (MACS) in combination with fluorescence-activated cell sorting (FACS). Single-cell suspensions of the brain tissue were prepared as described above (“Flow cytometry” section). Cells were stained with APC-conjugated anti-mouse CD45 antibody and PE-conjugated anti-mouse CD11b for 30 min at 4 °C. Unstained antibody was washed in PBS and cells were incubated with anti-PE microbeads at 4 °C for 15 min. HBSS was used to wash off unlabeled microbeads. Cells labeled with primary antibody conjugated to PE were enriched by using MACS columns (Miltenyi Biotech, Bergisch Gladbach Germany) according to the explanatory memorandum, and then targeted microglia were gathered using the FACS Aria cell sorting system. Resident microglia were identified as the CD45^int^CD11b^+^ population, whereas infiltrated macrophages in the CNS were identified as the CD11b^+^CD45^high^F4/80^+^ population. In addition, cell sorting of macrophages from the spleen was performed following the method in the “Flow cytometry” section to obtain the cell suspensions of the spleen. Then, cells were stained with APC-conjugated anti-mouse CD45 antibody, PE-conjugated anti-mouse CD11b, and FITC-conjugated anti-mouse F4/80 30 min at 4 °C. Unstained antibody was washed in PBS, and then targeted macrophages defined as the CD45^high^CD11b^+^ F4/80^+^ population were gathered using the FACS Aria cell sorting system. Isolated cells were collected in trizol reagent, vortexed, and kept at − 80 °C for further experiments.

### Isolation of primary microglia

Primary rat microglia culture was isolated as previously reported [35]. In brief, the cerebral cortices separated from neonatally 1-day-old rats and meninges were removed. Trypsinization was used to digest the striped cortical tissues for 30 min, and 70-μm nylon mesh cell strainer was used to obtain the mixed cortical cells. Cells were maintained in DMEM/F12 with fetal bovine serum (FBS), penicillin, and streptomycin (Gibco, Grand Island, NY, USA). Culture media were changed every 3 days until achieving a confluent monolayer at approximately 15 days. For the isolation of primary microglia, mild trypsinization was added to isolate microglia from the mixed glial cells. Purified microglia were cultured at 37 °C under atmosphere condition for further experiments.

### Oxygen-glucose deprivation (OGD)

To establish an ischemic-like condition in vitro, primary microglia were subjected to OGD as previously reported [36]. Briefly, microglia were cultured with serum-glucose-deprived cultures and placed in a hypoxic chamber with 95% nitrogen and 5% CO_2_ for 5 min and sealed tightly. Subsequently, the chamber moved to an incubator under 5% CO_2_/37 °C for 3 h. After the OGD treatment, serum and glucose-free medium were exchanged by glucose-containing medium with or without rhFGF21, which was followed by incubating with 95% air and 5% CO_2_ for 5 h and then analysis by qRT-PCR.

### Cell culture and treatment

Primary cultured microglia and the BV2 cell line were used to characterize the effect of rhFGF21 on microglial polarization, inflammation cytokine release, and NF-κB and PPAR-γ signaling activation. Cells were exposed to lipopolysaccharide (LPS) to induce polarized microglia and inflammatory secretion [37]. Briefly, microglia were treated with LPS (250 ng/mL) in the presence and absence of rhFGF21 (100 nM) or PD173074 (10 μM) for 4 h. Gene assays (involving IL-1β, iNOS, TNF-α, IL-6, CD86, CD206, Arg-1, IGF-1, and IL-10) were then detected in LPS-stimulated or OGD-treated primary microglia by qRT-PCR, and the effects of rhFGF21 on transcriptional activity of NF-κB in primary microglia were detected using immunofluorescence. Additionally, the polarization of microglia was analyzed by assessing the expression of the M1 marker CD86, which was identified by FACS staining.

### Western blot

Total proteins of LPS-treated BV2 cells were purified by RIPA lysis supplemented with a protease and phosphatase inhibitor mixture. Protein concentrations were measured with a Bradford Protein Detection Kit. Then, 60 μg of proteins from the samples and positive controls were separated on sodium dodecyl sulfate (SDS) polyacrylamide gels by electrophoresis. Subsequently, proteins were transferred onto PVDF membranes followed by blocking with primary antibodies FGFR1 (1:1000), p-FGFR1 (1:1000), NF-κB (1:1000), PPAR-γ (1:400), and β-Actin (1:500) overnight at 4 °C. Then, the membranes were incubated with secondary antibody donkey anti-rabbit IgG or goat anti-mouse IgG at a 1:10,000 dilution for 1 h at room temperature. Finally, the protein bands were detected with Image Lab software using Gel Doc Imager (Bio-Rad, Hercules, CA, USA) and the expression of target proteins was normalized against β-Actin.

### Immunofluorescence analysis

Immunofluorescence staining was performed on paraffin brain sections as previously described [38]. Briefly, non-specific binding of antibodies was blocked with 5% BAS for 1 h at 37 °C and the sections were then incubated with one or more primary antibodies against CD16/32, CD206, Iba1, or NF-κB in a dilution following the manufacturer’s instruction at 4 °C overnight. After washing, secondary antibodies conjugated with adequate fluorochrome were added to visualize the expression of corresponding proteins and DAPI was used to stain the nuclei. Images of the penumbra of the infarct cortex were captured using a confocal laser scanning microscope (Laika, Japan). Data were analyzed with ImageJ (NIH Image, Bethesda, MD, USA) to calculate the fluorescence intensity or counting number of recognized cells per field.

### Statistical analysis

All statistical analyses of the data were processed with Prism 7.0 software (GraphPad, San Diego, CA, USA) in a blinded manner. Data from individual groups were expressed as mean ± SEM and characterized by a one-way ANOVA for multiple comparisons or Student’s *t* test (and nonparametric tests). Behavioral data were statistically analyzed by a two-way ANOVA for multiple comparisons. Statistical significance was considered at *P* < 0.05 level.

## Results

### rhFGF21 protects against brain injury in MCAO mice

**Fig. 1:**
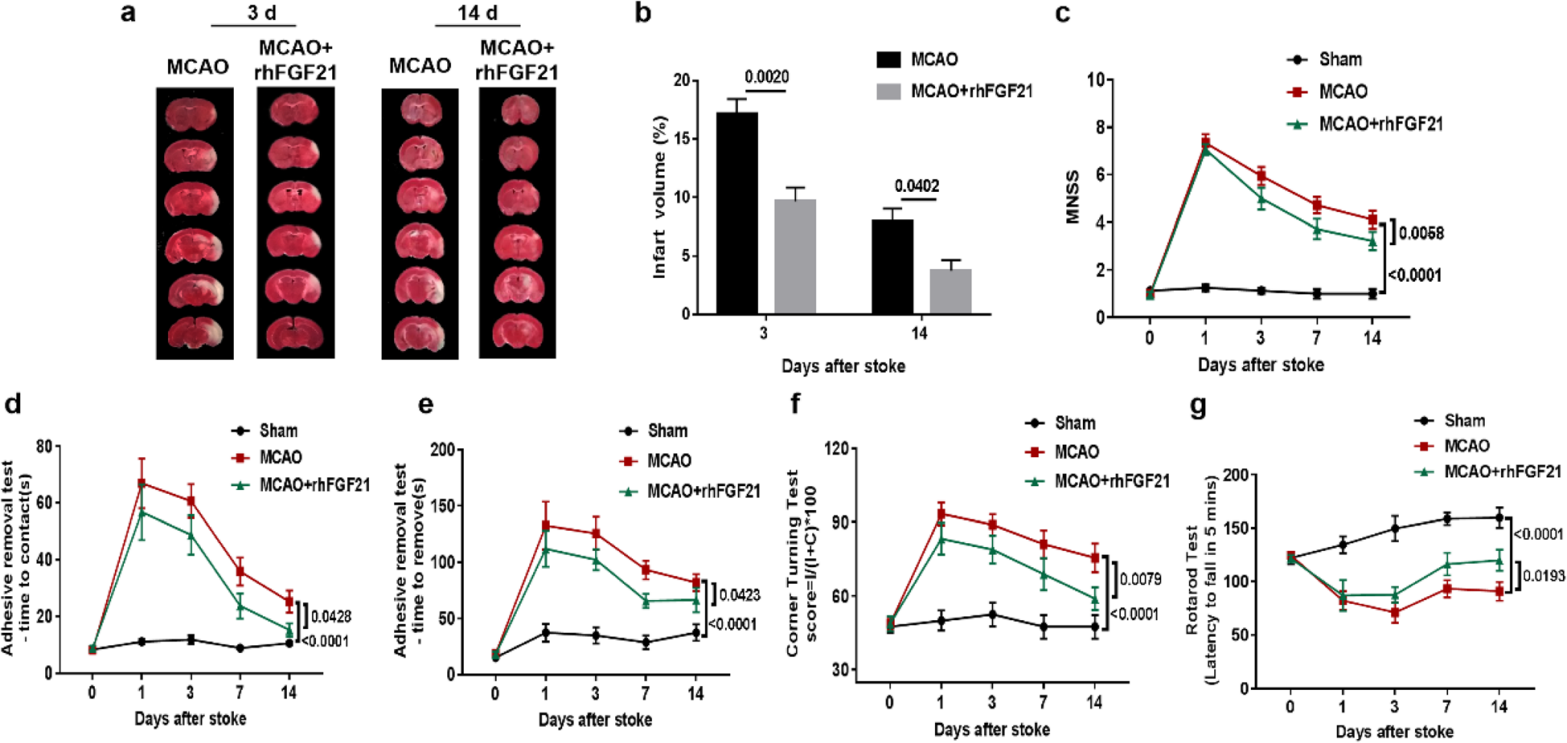
Effects of rhFGF21 on infarct volumes and neurodeficits in the MCAO model. **a** Representative sample of brain slices with 2% TTC staining at 3 and 14 days after MCAO, and **b** quantification of the significant difference in the figure. *n* = 8. Values are mean ± SEM by unpaired *t* tests. **c** mNSS assessed at 1, 3, 7, and 14 days after MCAO. **d**–**g** Cumulative data illustrate the indicated neurobehavioral tests, including the time to contact (**d**) and the time to removal in the adhesive test (**e**) and results from the corner-turning test (**f**) and rotarod test (**g**) from day 1 to 14 after MCAO. *n* = 4 in the sham group, *n* = 10 in the MCAO and rhFGF21-treated group. Values are mean ± SEM by two-way ANOVA

Previous reports have shown that rhFGF21 significantly reduces infarct volumes at 24 h after focal ischemia in rats compared with the vehicle treatment [39]. To assess the effect of rhFGF21 on brain injury after ischemic stroke in mice, infarct volumes were detected at 3 and 14 days after MCAO. The decreased infarct volume reached significance at 3 days after MCAO based on 2,3,5-triphenyltetrazolium chloride (TTC) staining, and rhFGF21 also significantly reduced the infarct size at 14 days after MCAO (Fig. 1a). The percentage of infarct volume in the MCAO group gradually decreased from 17.16 ± 1.277 at 3 days after MCAO to 9.657 ± 1.189 at 14 days after MCAO, and this percentage in the MCAO+rhFGF21 group decreased from 7.971 ± 1.077 to 3.744 ± 0.913 (Fig. 1b). Moreover, behavioral assessments were evaluated at 1, 3, 7, and 14 days after MCAO. Neurological deficits and feeling function in the rhFGF21 group, assessed by the modified neurological severity score (mNSS) test (Fig. 1c) and removal test (Fig. 1d, e), respectively, were significantly better than those in the MCAO group. Similarly, the rhFGF21-treated group exhibited a significant improvement in sensorimotor function, demonstrated by fewer right turns in the corner-turning test (Fig. 1f), and enhanced motor coordination, indicated by an increased latency to fall off the rotarod (Fig. 1g), compared to the vehicle-treated group at 14 days after MCAO. Together, these behavioral data suggest that post-stroke treatment with rhFGF21 facilitates functional recovery after MCAO.

### rhFGF21 inhibits the inflammatory response in the cortex of the ischemic brain

**Fig. 2:**
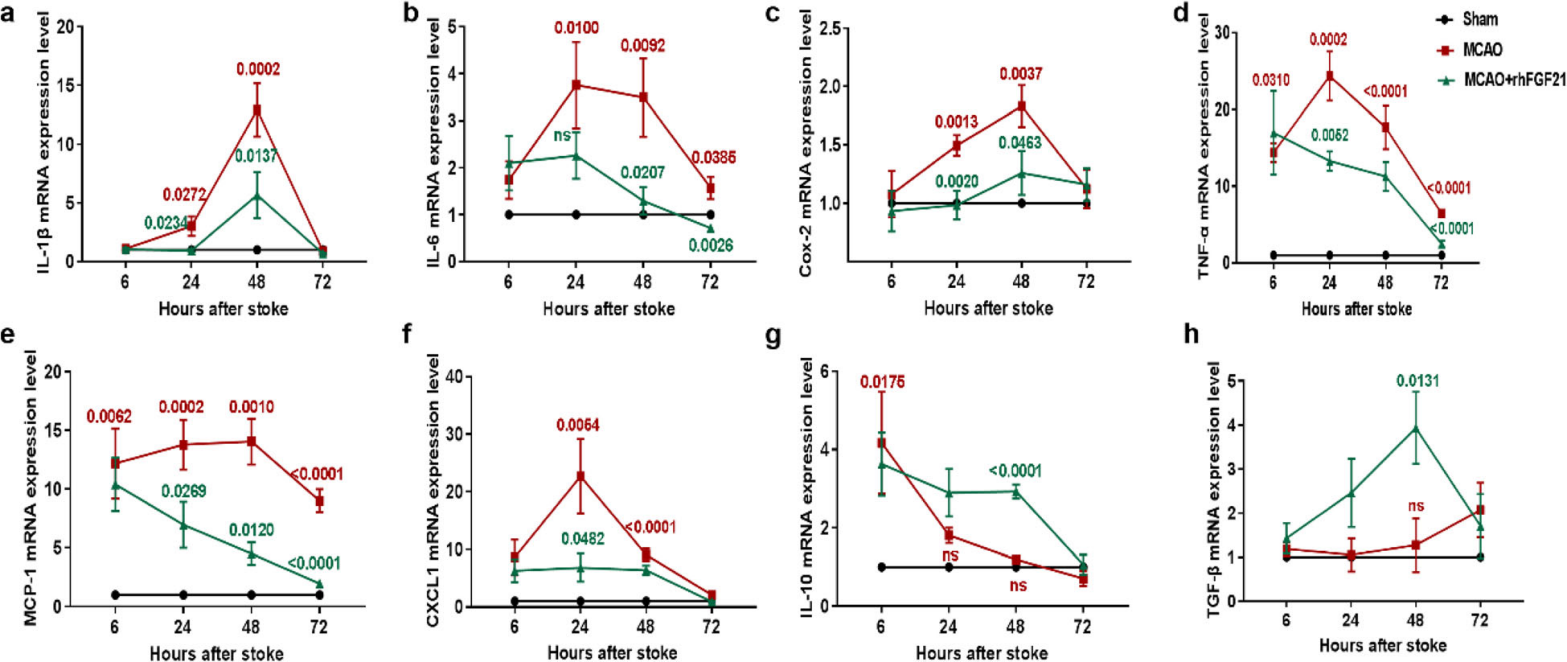
Effects of rhFGF21 on inflammatory cytokines after MCAO. mRNA expression levels of IL-1β (**a**), IL-6 (**b**), COX-2 (**c**), TNF-α (**d**), MCP-1 (**e**), CXCL1 (**f**), IL-10 (**g**), and TGF-β (**h**) in the cortex around the infarcted zone were detected via qRT-PCR at 6, 24, 48, and 72 h after MCAO. Values are mean ± SEM by one-way ANOVA, *n* = 6 per group. *p* value (red) of MCAO versus sham, *p* value (green) of MCAO versus MCAO+rhFGF21

The secretion of inflammatory cytokines plays a unique role in the inflammatory cascade reaction and neuronal injury following stroke. In this study, an array of inflammatory cytokines including IL-1β, TNF-α, IL-6, cyclooxygenase (COX)-2, monocyte chemoattractant protein (MCP)-1, and chemokine (C-X-C motif) ligand 1 (CXCL1) at 6, 24, 48, and 72 h after stroke were analyzed by qRT-PCR. mRNA expression of pro-inflammatory cytokines, including IL-6, TNF-α, and CXCL1, were quickly increased after stroke and peaked at 24 h (Fig. 2b, d), whereas IL-1β, COX-2, and MCP-1 levels peaked at 48 h (Fig. 2a, c, e) after stroke. However, rhFGF21 administration markedly suppressed the stroke-evoked enhancement of cytokine levels beginning at 24 h, especially at 24 and 48 h after stroke (Fig. 2a–f). Interestingly, levels of IL-10, generally regarded as an anti-inflammatory cytokine, were robustly but transiently increased at 6 h after stroke; however, this high level of expression continued to 48 h in the rhFGF21 treatment groups (Fig. 2g). Furthermore, compared to vehicle, rhFGF21 significantly elevated the level of TGF-β at 48 h (Fig. 2h). These results suggest that rhFGF21 ameliorates the MCAO-induced inflammatory response at the acute stage.

Additionally, IL-10, as an M2 marker, is commonly considered to be a mediator of microglial phenotype polarization [38], and TNF-α, IL-1β, IL-6, and MCP-1 are secreted by M1 microglia. Therefore, we speculate that rhFGF21 may affect microglia and their polarization.

### rhFGF21 modulates microglia polarization and the temporal presence of microglial phenotypes and number in the ischemic brain

**Fig. 3:**
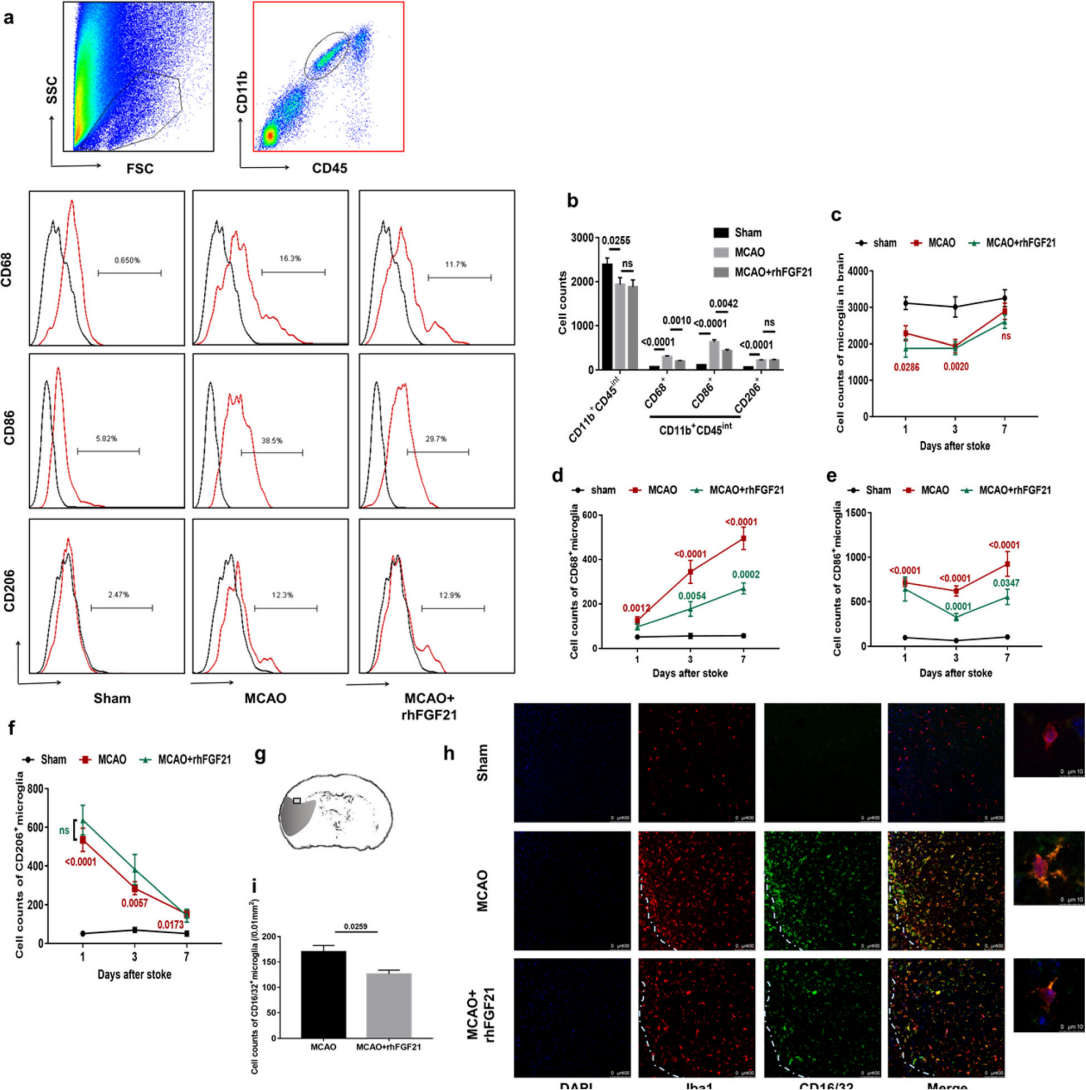
Effects of rhFGF21 on microglial polarization. **a** Representative pseudocolor and histograms of flow cytometry show the gating strategy for microglia (CD11b^+^CD45^int^) and CD68^+^, CD86^+^, and CD206^+^ expressing microglia in cell suspensions from ischemic hemispheres. All gates were set using FMO control samples. **b** Bar graph summarizing the cell counts of microglia (CD11b^+^CD45^int^) and CD68^+^, CD86^+^, and CD206^+^ expressing microglia in the brain 3 days after MCAO. **c**–**f** Quantification of flow cytometry shows the number of microglia (**c**) and their expression of CD68 (**d**), CD86 (**e**), and CD206 (**f**) at 1, 3, and 7 days. **g** Sketch picture indicating the area of the immunofluorescence pictures obtained from the penumbra of the infarct cortex. **h** Immunofluorescence staining shows CD16/32 expression by microglia (Iba-1) in the peri-infarct area at 3 days after MCAO under confocal observation. **i** Quantification of the counts of CD16/32^+^ microglia. *n* = 6 in sham the group, *n* = 12 in the MCAO and rhFGF21-treated group. Values are mean ± SEM, *p* value (red) of MCAO versus sham, *p* value (green) of MCAO versus MCAO+rhFGF21

To investigate the potential impact of rhFGF21 on microglia in the brain after focal ischemic stroke, we first measured the number and phenotypes of resident microglia in the ischemic hemisphere by flow cytometry analysis at 3 days after stroke. Resident microglia were defined as CD11b^+^CD45^int^ cells, and the populations of CD68, CD86, and CD206 microglia were gated using FMO controls (Fig. 3a). The MCAO group had significantly fewer microglia than the sham group, although there were no significant differences in the count of microglia between the vehicle-treated group and rhFGF21-treated group. Moreover, the counts of CD68^+^, CD86^+^, and CD206^+^ microglia in the ischemic hemisphere were significantly increased in the MCAO group compared with the sham group, and rhFGF21 obviously suppressed the expression of cell counts of the CD68^+^ and CD86^+^ microglia evoked by MCAO but did not markedly affect the variation of cell counts of CD206^+^ microglia (Fig. 3b).

The distribution of the microglial phenotype undergoes dynamic changes depending on timing and context; therefore, we further analyzed the variation in microglial phenotype at 1, 3, and 7 days after MCAO. The number of resident microglia was markedly lower than that in the sham group from 1 to 3 days after stroke; however, this returned to a similar level as that in the sham group by day 7 (Fig. 3c). Furthermore, the expression of CD68 was significantly upregulated beginning at 1 day following the ischemic event and continuing until 7 days post-stroke. Importantly, rhFGF21 intervention significantly reversed the elevated CD68 expression, most strongly at 3 days and 7 days after stroke (Fig. 3d). CD86 has been classified as an M1 and M2b microglia marker that reduces the polarization of microglia to the M2a phenotype [37]. CD86 expression was significantly higher than that in the sham group at 24 h after MCAO but then decreased at day 3. However, the protein level of CD86 peaked at 7 days after MCAO at a level higher than that expressed at the other time points. Moreover, exposure to rhFGF21 treatment markedly suppressed the elevation of CD86 at 3 and 7 days after MCAO (Fig. 3e**)**. Intriguingly, the number of CD206^+^ microglia transiently increased in the injured brain as early as 1 day after MCAO and then gradually decreased, consistent with a previous report [11]. However, compared with vehicle treatment, rhFGF21 did not significantly affect the expression of CD206 in microglia (Fig. 3f).

In addition, an immunohistochemistry approach was used to assess the expression of CD16/32, another marker of M1 microglia, in the penumbra of the infarct cortex and corresponding cortical tissue of animals at 3 days after MCAO (Fig. 3g). Consistently, the rhFGF21 treatment obviously attenuated the high expression of CD16/32 (Fig. 3h, i). Collectively, these findings suggest that post-stroke treatment with rhFGF21 inhibits the polarization of M1 microglia but does not facilitate the polarization of microglia toward the M2 phenotype.

### rhFGF21 reduces immune cell infiltration in the CNS and M1 macrophage accumulation

**Fig. 4:**
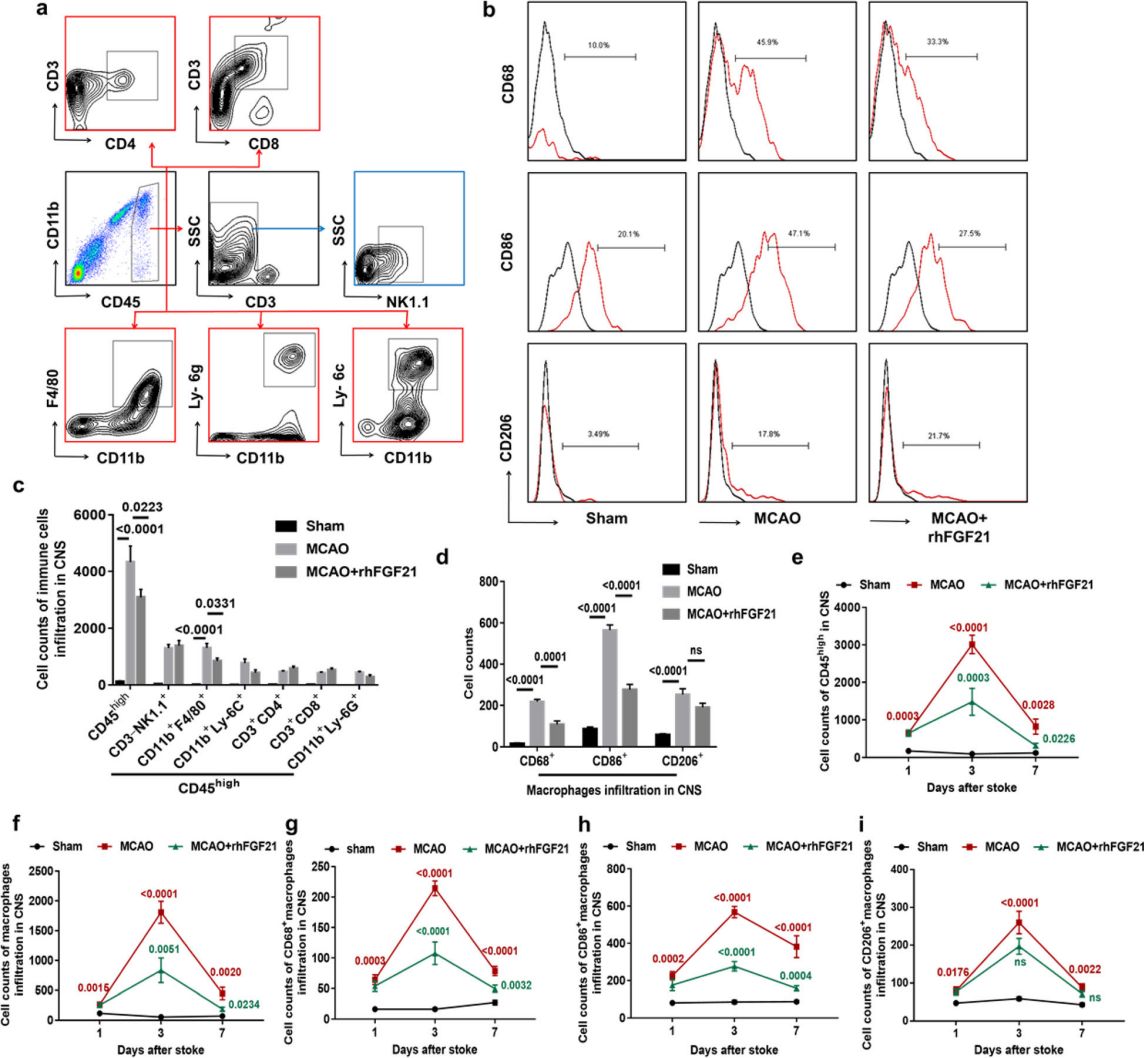
Effects of rhFGF21 on migrated peripheral immune cell infiltration in the CNS. **a** Representative flow cytometry analysis shows the gating strategy for NK cells (CD45^high^CD3^-^NK1.1^+^), CD4^+^T cells (CD45^high^CD3^+^CD4^+^), CD8^+^T cells (CD45^high^ CD3^+^CD8^+^), macrophages (CD11b^+^CD45^high^F4/80^+^), neutrophils (CD11b^+^CD45^high^Ly-6G^+^), and monocyte cells (CD11b^+^CD45^high^Ly-6G^−^Ly-6C^+^) using FMO. **b** Gating strategy of CD68, CD86, and CD206 from the subsets of macrophages using FMO control. **c** Quantification analysis shows the cumulative data for migrated peripheral immune cells at 3 days post-stroke. **d** Protein levels of CD68, CD86, and CD206 expressed in macrophages. **e**, **f** Line graphs summarize the flow cytometry data showing the dynamic distribution of infiltrated immune cells defined as CD45^high^ (**e**) and macrophages (**f**) in the brain at 1, 3, and 7 days after stroke. **g**–**i** Temporal presence of CD68^+^ (**g**), CD86^+^ (**h**), and CD206^+^ (**i**) macrophages at 1, 3, and 7 days after stroke among the three groups. *n* = 6 in the sham group, *n* = 12 in the MCAO and rhFGF21-treated group. Values are mean ± SEM by one-way ANOVA. *p* value (red) of MCAO versus sham, *p* value (green) of MCAO versus MCAO+rhFGF21

Subsequently, we analyzed the accumulation of infiltrating immune cells defined as CD45^high^ cells and the infiltration of NK (CD45^high^CD3^−^NK1.1^+^), CD4^+^T (CD45^high^CD3^+^CD4^+^), CD8^+^T (CD45^high^CD3^+^CD8^+^), neutrophils (CD11b^+^CD45^high^ Ly-6G^+^), macrophages (CD11b^+^CD45^high^F4/80^+^), and monocyte cells (CD11b^+^ CD45^high^Ly-6C^+^) in the CNS at 3 days after MCAO (Fig. 4a). Flow cytometry analysis revealed that rhFGF21 administration did not significantly affect the numbers of NK, CD4^+^T, and CD8^+^T cells that infiltrated in the CNS. However, rhFGF21 treatment of MCAO animals strikingly reduced the number of infiltrating immune cells defined as CD45^high^ cells and macrophages (CD11b^+^CD45^high^F4/80^+^) compared with the vehicle treatment (Fig. 4c). Meanwhile, the number of neutrophils (CD11b^+^CD45^high^ Ly-6G^+^) and monocyte cells (CD11b^+^ CD45^high^Ly-6C^+^) in the rhFGF21-treated MCAO group was slightly lower than that in the vehicle-treated MCAO group.

In addition, the phenotype of infiltrating macrophages gated on CD11b^+^CD45^high^ F4/80^+^ was further detected by flow cytometry analysis at 3 days following stroke (Fig. 4b). The counts of CD68^+^ and CD86^+^ macrophages were significantly lower in rhFGF21-treated MCAO mice than in the vehicle-treated MCAO mice, although there were no significant differences in the count of CD206^+^ macrophages between the vehicle-treated and rhFGF21-treated groups (Fig. 4d).

Moreover, we further detected the temporal profiles when infiltrating immune cells defined as CD45^high^ cells, macrophages, and CD68^+^, CD86^+^, and CD206^+^ macrophages were present in the ischemic brain at 1, 3, and 7 days following stroke. The absolute counts of infiltrated immune cells (Fig. 4e), macrophages (Fig. 4f), and CD68^+^, CD86^+^, and CD206^+^ macrophages (Fig. 4g–i) were dramatically increased in the CNS of MCAO mice, and they peaked at 3 days post-injury and subsequently returned to baseline levels by 7 days. Similar temporal profiles were also observed in rhFGF21-treated MCAO mice, but the cell counts of infiltrated immune cells (Fig. 4e), macrophages (Fig. 4f), and CD68^+^ and CD86^+^ macrophages (Fig. 4g, h) that accumulated in CNS were significantly decreased in rhFGF21-treated MCAO mice compared with the vehicle-treated MCAO mice at 3 days and 7 days after stroke. Together, these results together suggest that rhFGF21 alleviates the accumulation of infiltrated immune cells (particularly macrophages) in the ischemic brain and might contribute to the inhibition of the macrophage-mediated inflammatory response.

### rhFGF21 suppresses the phenotypic alteration of macrophages toward the M1 in the spleen and blood

**Fig. 5:**
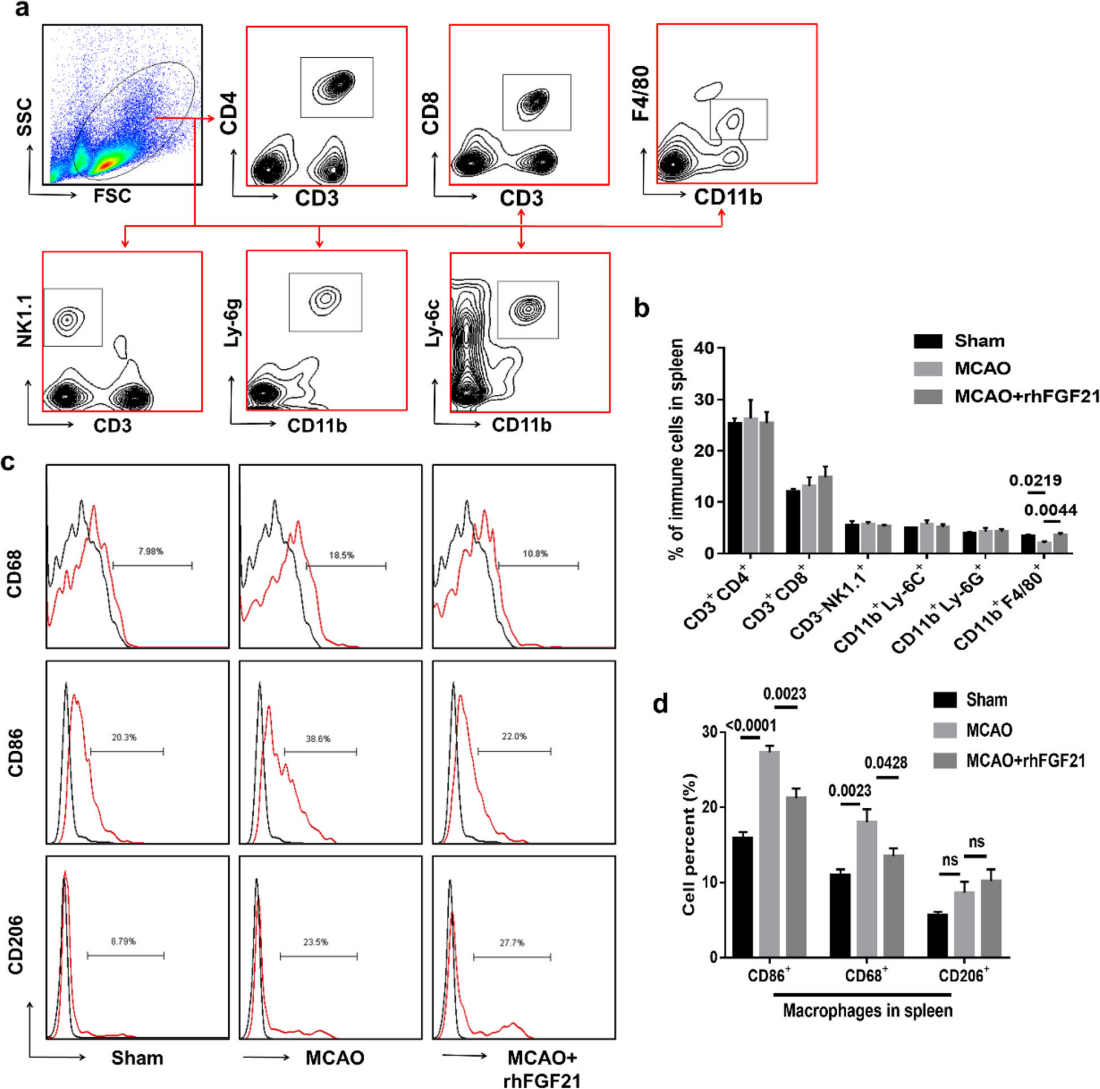
Effects of rhFGF21 on peripheral immune cells in the spleen at day 3 after MCAO in mice. **a** Representative gating strategy of neutrophils (CD11b^+^Ly6G^+^), monocytes (CD11b^+^Ly6C^+^), CD8^+^ T cells, CD4^+^ cells, NK1.1^+^ cells, and macrophages (CD11b^+^F4/80^+^) in a single-cell suspension from the spleen using FMO control samples. **b** Cumulative data for quantifying the percentage of the above immune cell subsets. **c** Gating strategy of CD68, CD86, and CD206 expressed in macrophages from the spleen 3 days after stroke. **d** Bar graph shows the percentage of CD68^+^, CD86^+^, and CD206^+^ cells in macrophages. *n* = 6 in the sham group, *n* = 10 in the MCAO and rhFGF21-treated group. Values are mean ± SEM by one-way ANOVA

**Fig. 6:**
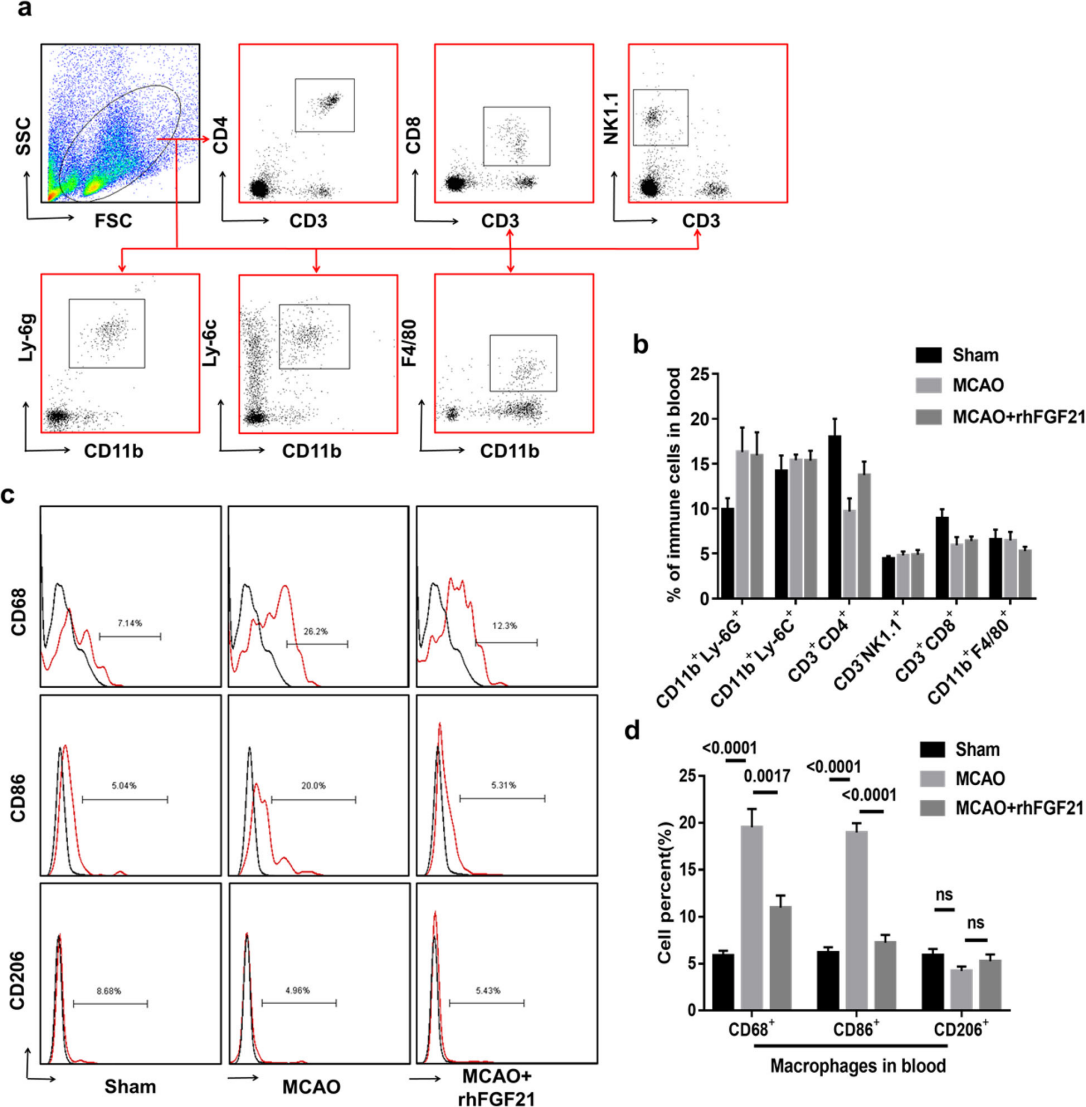
Effect of rhFGF21 on immune cells from blood at 3 days after MCAO. **a** Representative dot plot showing the gating strategy of immune cell subsets from the blood. **b** Quantification analysis shows the percentage of NK cells, CD4^+^ T cells, CD8^+^ T cells, macrophages, neutrophils, and monocytes in the blood. **c** Gating strategy of CD68, CD86, and CD206 in macrophages. **d** Summarized flow cytometry data for quantifying CD68 and CD86 and CD206 expression in macrophages from the blood. *n* = 6 in the sham group, *n* = 10 in the MCAO and rhFGF21-treated group. Values are mean ± SEM by one-way ANOVA

Excessive stroke-induced immune responses lead to disturbances in peripheral immunity, which in turn interfere with immune cell infiltration in the injured brain [13]. rhFGF21 has been reported to inhibit macrophage-mediated inflammation by suppressing NF-κB in RAW 264.7 cells [29]. To determine whether rhFGF21 alleviates CNS inflammation mediated by macrophage-mediated peripheral inflammation, we performed a flow cytometry analysis to detect alterations in peripheral immune cell subsets in the spleen and blood at 3 days post-stroke. The gating strategies of neutrophils (CD11b^+^Ly6G^+^), monocytes (CD11b^+^Ly6C^+^), CD8^+^ T cells, CD4^+^ cells, NK1.1^+^ cells, and macrophages (CD11b^+^F4/80^+^) in single-cell suspensions from the spleen (Fig. 5a) and blood (Fig. 6a) were set by FMO control, and the gate settings of CD68^+^, CD86^+^, and CD206^+^ macrophages were set by FMO control in the spleen (Fig. 5c) and blood (Fig. 6c). After MCAO, the number of macrophages in the peripheral spleen organs was substantially diminished (Fig. 5b), although there was no significant difference in the blood (Fig. 6b). Notably, variation in other cell subsets was not observed in either the spleen or the blood. In contrast, the depletion of macrophages induced by MCAO was effectively alleviated by rhFGF21. Moreover, rhFGF21 significantly rescued the increased expression of CD68 and CD86 in macrophage cells residing in the spleen (Fig. 5d) and blood (Fig. 6d) following MCAO. However, there was no considerable difference in the expression of CD206 in the macrophages between the vehicle- and rhFGF21-treated groups. These results suggest that rhFGF21 reduces macrophage activation in peripheral tissue, which is associated with the inflammatory processes in the CNS.

### rhFGF21 regulates the secretion of IL-1β, TNF-α, IL-6, and TGF-β cytokines in sorted microglia and macrophages at 3 days after stroke

**Fig. 7:**
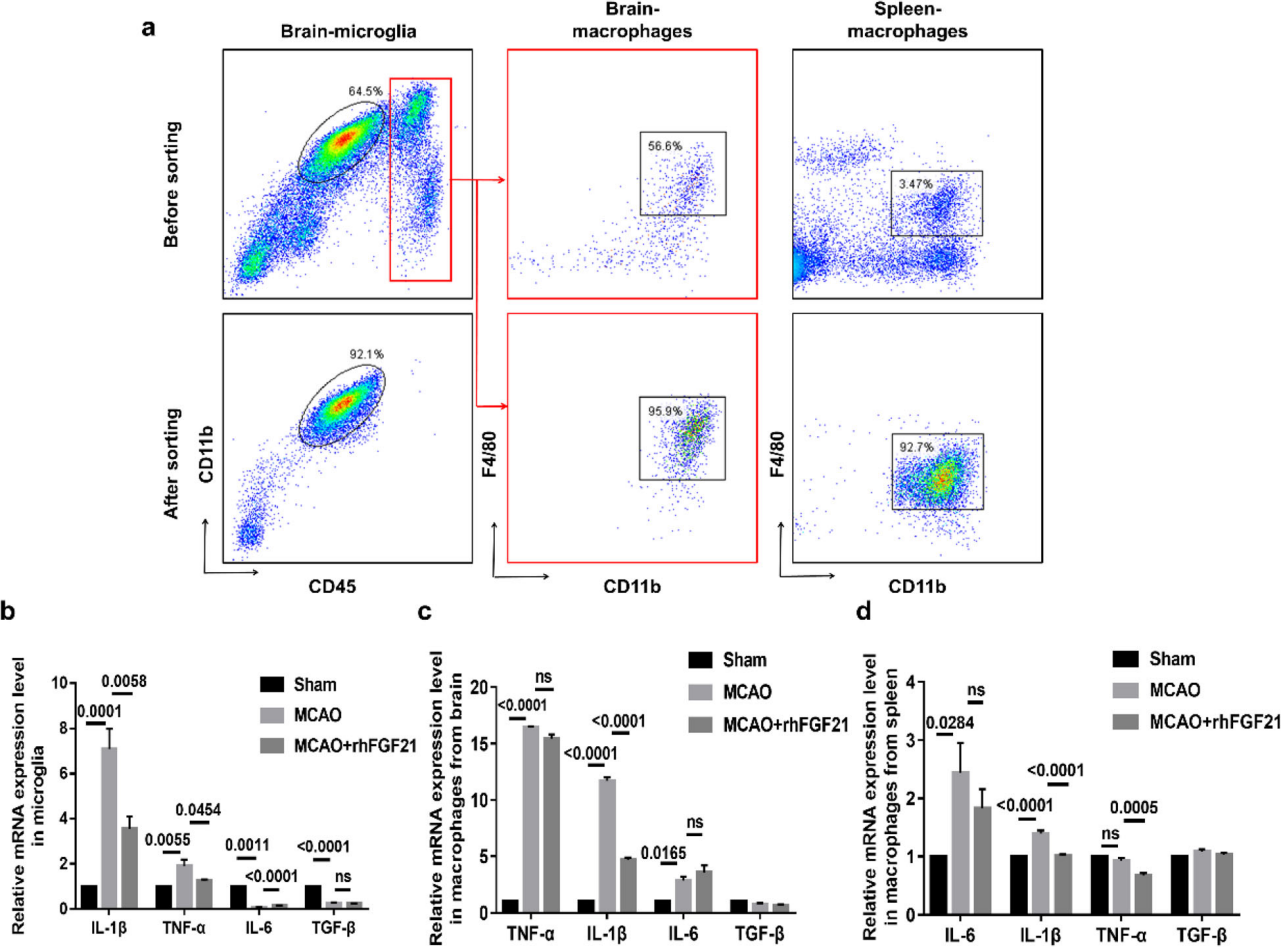
Effects of FGF21 on inflammatory cytokines (IL-1β, TNF-α, IL-6, and TGF-β) in sorted microglia and macrophages. **a** Microglia (CD11b^+^CD45^int^) from the brain, and macrophages (CD11b^+^CD45^high^F4/80^+^) from the brain or spleen sorted by FACS coupled with MACS, producing a purity of above 90%. **b**–**d** qRT-PCR shows the gene expression of IL-1β, TNF-α, IL-6, and TGF-β in microglia (**b**) and macrophages from the brain (**c**) and macrophages from the spleen (**d**). *n* = 8 per group. Values are mean ± SEM by one-way ANOVA

To further detect the potential effects of rhFGF21 on the release of inflammatory cytokines in microglia and macrophages, we sorted microglia and infiltrated macrophages from the damaged hemisphere, and macrophages from the spleen to a purity above 90% and assessed gene expression by real-time PCR (Fig. 7a). Compared with the levels in the sham group, the expression levels of IL-1β (5.33-fold) and TNF-α (1.42-fold) in microglia in the MCAO group were dramatically increased, but those of IL-6 (0.09-fold) and TGF-β (0.28-fold) were significantly reduced. However, both the enhancement in IL-1β and TNF-α expression and the decline in IL-6 expression were hindered by the administration of rhFGF21, but TGF-β expression was not significantly affected (Fig. 7b). In addition, upon induction of MCAO, the mRNA levels of IL-1β (11.70-fold), TNF-α (16.36-fold), and IL-6 (2.87-fold) in infiltrated macrophages were dramatically higher than those in macrophages from the spleen. In the presence of rhFGF21, IL-1β expression was remarkably inhibited, but TNF-α and IL-6 expression levels were not significantly affected. In contrast, there were no differences in TGF-β expression among all groups (Fig. 7c). Moreover, administration of rhFGF21 effectively attenuated the MCAO-induced increase in the levels of IL-1β (1.40-fold) and TNF-α (0.93-fold) in macrophages from the spleen but did not remarkably affect the levels of IL-6 (2.44-fold) or TGF-β (1.08-fold) following MCAO (Fig. 7d). In summary, these outcomes further indicate that rhFGF21 mediates anti-inflammatory effects by modulating microglia and macrophages.

### rhFGF21 reduces M1 marker expression and pro-inflammatory cytokine secretion in primary microglia treated with oxygen-glucose deprivation (OGD) or lipopolysaccharide (LPS)

**Fig. 8:**
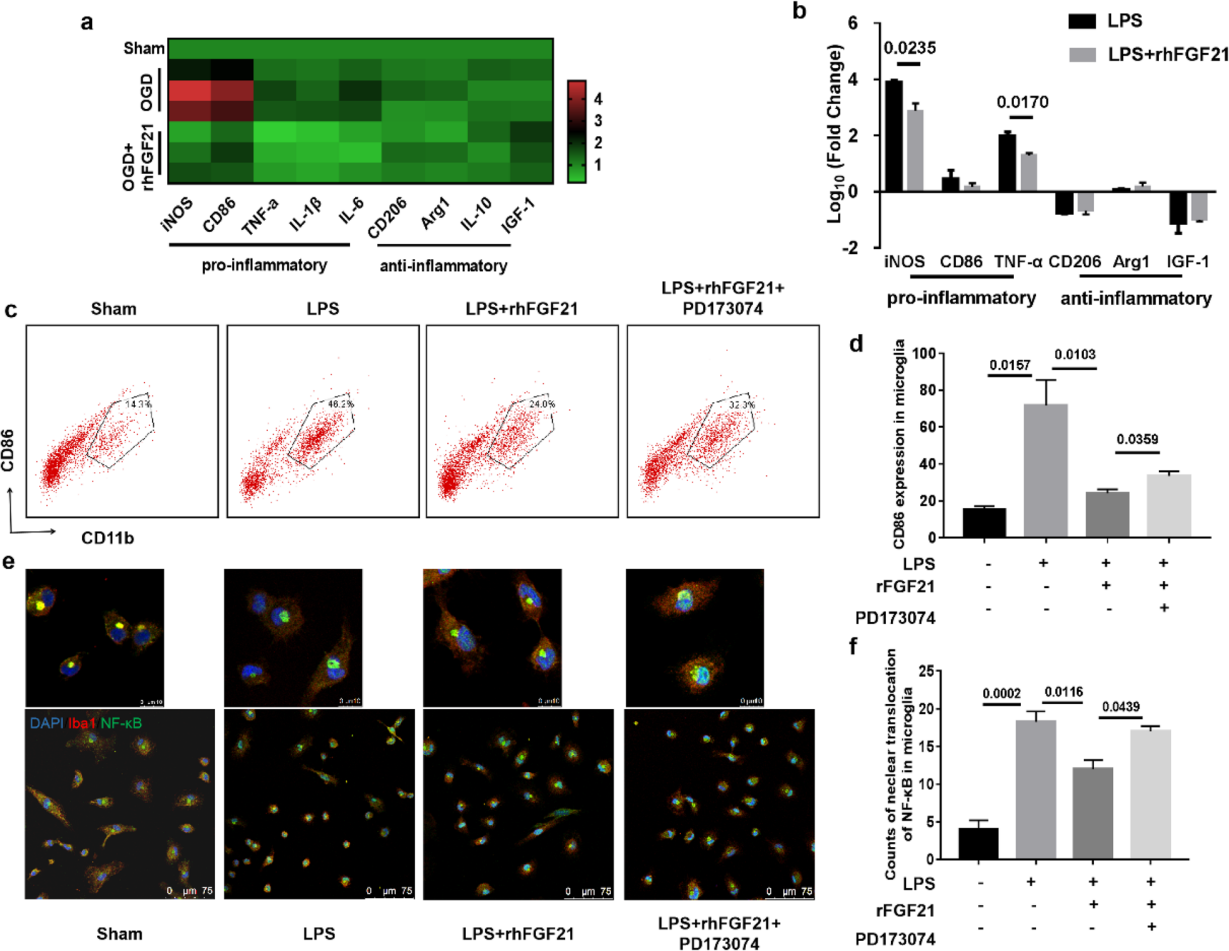
Effect of rhFGF21 on the inflammatory response in primary microglia in vitro. **a**, **b** Primary cultured microglia were exposed to OGD (**a**) or LPS (**b**) plus vehicle or rhFGF21 and subsequently subjected to qRT-PCR to detect the gene expression of pro-inflammatory molecules iNOS, CD86, TNF-α, IL-1β, and IL-6 and anti-inflammatory molecules CD206, Arg-1, IL-10, and IGF-1. **c**, **d** Dot plots (**c**) and summarized graph (**d**) based on the flow cytometry results show the expression of CD86 in primary microglia evoked by LPS. **e** Colocalization of NF-κB (green) with nuclei (blue) in Iba1 (red)-marked microglia revealed that the nuclear translocation of NF-κB was blocked by rhFGF21 but that the influence of rhFGF21 was reversed by PD173074. **f** Statistical analysis shows the counts of microglia in which NF-κB translocate into the nuclei. *n* = 4 per group. Values are mean ± SEM by one-way ANOVA

To evaluate the effects of rhFGF21 on the polarization of microglia and pro-inflammatory cytokine production, we examined primary microglia stimulated by OGD or LPS. Similar results were observed, the administration of rhFGF21 markedly suppressed the expression of M1-type genes (IL-1β, iNOS, TNF-α, IL-6, and CD86), but did not affect the M2-type genes (CD206, Arg-1, IGF-1, and IL-10) in OGD-treated primary microglia (Fig. 8a). Similarly, rhFGF21 markedly inhibited the mRNA level of iNOS and TNF-α in LPS-stimulated primary microglia (Fig. 8b), and the protein expression of CD86 was detected at the protein level using flow cytometry assays (Fig. 8c, d). Moreover, microglial activation during ischemic injury along with the activation of NF-κB is associated with the secretion of inflammatory cytokines. As shown in Fig. 8e, immunofluorescent staining (× 400) demonstrated that rhFGF21 suppressed the nuclear translocation of NF-κB, indicating that the transphosphorylation activity of NF-κB was inhibited by rhFGF21. However, co-administration with PD173074, a selective inhibiter of FGFR1, reversed this effect of rhFGF21. Moreover, the counts of microglia for which NF-κB is translocated into the nuclei are quantified in Fig. 8f, which indicates that the transcriptional activity of NF-kB was suppressed by rhFGF21. These data together demonstrate that rhFGF21 ameliorates microglia-mediated neuroinflammation by inhibiting NF-κB signaling via the FGFR1 receptor.

### rhFGF21 upregulates PPAR-γ and inhibits the activation of NF-κB via FGFR1 in LPS-stimulated BV2 cells

**Fig. 9:**
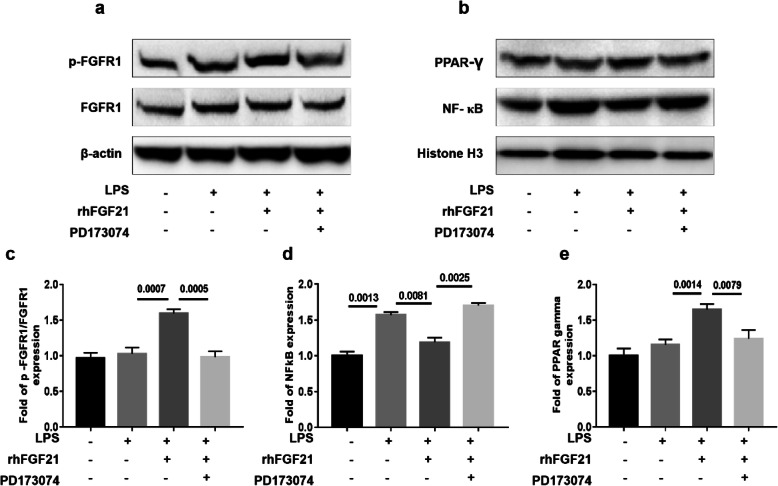
Effects of rhFGF21 on PPAR-γ and NF-κB signaling in LPS-stimulated BV2 cell line. **a** Representative band of p-FGFR1 and FGFR1 by western blot assay based on an internal control of β-actin and quantitated in the graph (**c**). **b** Amount of NF-κB and PPAR-γ in the nucleus of the control with H3 and quantitated in the graph (**d** NF-κB and **e** PPAR-γ). *n* = 4 per group. Values are mean ± SEM by one-way ANOVA

NF-κB activation in microglia is associated with pro-inflammation, whereas the activity of PPAR-γ is positively related to anti-inflammation. To evaluate whether rhFGF21 activated PPAR-γ and inhibited NF-κB, we performed western blot experiments in BV2 cells exposed to LPS. As expected, rhFGF21 significantly upregulated the phosphorylation level of FGFR1; however, PD173074 obviously reserved this upregulation (Fig. 9a, c). In addition, similar to observations in primary microglia, rhFGF21 markedly suppressed the transcriptional activity of NF-κB, which was reversed by PD173070 (Fig. 9b, d). Notably, rhFGF21 administration enhanced the expression of PPAR-γ (Fig. 9b, e), which commonly alters M2 gene expression. Additionally, the effect of rhFGF21 was reversed by PD173074. These data indicated that rhFGF21 modulates microglial polarization via the NF-κB and PPAR-γ pathways.

## Discussion

The inflammatory response evoked by focal ischemia stroke is a complex and pleiotropic process [13]. The complexity is emphasized by the immunomodulation progression associated with multiple immune cells—resident microglia and an influx of hematogenous cells, that changes based on the time, space, and stage-specific milieu. The heterogeneity is highlighted by detrimental and protective immune effects mediated by those immune cells. Regulation of neuroinflammation has been recognized as an attractive approach for promising therapies in stroke. rhFGF21 is a safe and effective endocrine regulator that has been demonstrated to have strong anti-inflammatory effects, and it represents a promising candidate for microglia/macrophage-based therapy in acute stroke.

In the current study, we demonstrated the neuroprotective effects of rhFGF21 and highlighted its immunomodulatory effects by regulating resident microglia and hematogenous macrophages in acute ischemic stroke. Although this study is not the first to show that rhFGF21 may protect against cerebral ischemic injury in rats [39], it confirmed that rhFGF21 significantly reduced the infarct size and ameliorated the neurological deficit in mice affected by stroke through a set of experiments (including TTC and behavior assessment). Moreover, our study also revealed that rhFGF21 remarkably dampened the upregulation of pro-inflammatory gene expression. Therefore, our study provides evidence that these outcomes associated with the neuroprotective effects of rhFGF21 are tightly linked with its anti-inflammatory function.

Post-ischemic inflammation is a hallmark of ischemic stroke pathology, which plays critical roles in acute brain damage and profoundly affects long-term recovery [40, 41]. Inflammation-associated conditions regulated by pro-inflammatory and anti-inflammatory cytokines are associated with impaired neurogenesis and neuronal survival [42]. Our findings are consistent with a previous report [28] that showed that almost all inflammatory cytokines were upregulated immediately following stroke, with levels peaking at 24 or 48 h after stroke. However, the increase in IL-10 expression appears strongly but transiently as early as 6 h post-stroke. Among the cytokines detected, TNF-α has both neurotoxic and neuroprotective effects, while IL-1β have characteristically neurotoxic effects. Both TNF-α and IL-1β are mainly produced by microglia and macrophages [43] and synthesized by segregated subsets [44]. The cellular source of IL-6 remains controversial, although it is likely predominantly expressed in threatened neurons and activated microglia around the infarct region and targeted at neurons or microglia, and it contributes to both the damage and repair processes [45]. TGF-β, as an anti-inflammatory cytokine, is associated with tissue repair. We next investigated the effect of rhFGF21 on inflammatory gene (TNF-α, IL-1β, IL-6, TGF-β) expression in microglia and invading peripheral macrophages, which are defined as the major cellular contributors to neuroinflammation [46, 47]. Although there are analogous phenotypic and functional characteristics among these inflammatory genes, numerous studies have revealed that they may play unique roles under pathological conditions [48]. Zarruk et al. [49] detected higher expression of TNF-α in microglia than in macrophages of LysM-EGFP knock-in mice and higher expression of IL-1β and Arg-1 in macrophages than in macroglia in a permanent MCAO model. In our model, rhFGF21 significantly reduced the level of IL-1β expression not only in microglia and infiltrated macrophages but also in splenic macrophages. Surprisingly, the mRNA level of IL-6 in resident microglia isolated from MCAO mice was far lower than that in sham mice. Although we have no suitable explanation for this phenomenon, investigating the temporal pattern of the source of IL-6 may provide a reasonable explanation.

Microglia are major cellular contributor to post-injury inflammation and have the potential to act as a key factor for disease onset and progression and contribute to the neurological outcome of acute brain injury [50]. Under pathological conditions, microglia are rapidly activated and undergo dramatic morphological and phenotypic changes accompanied by the induction of inflammatory cytokines. The classical activation phenotype (M1) is an inflammatory phenotype that produces pro-inflammatory cytokines, while the alternative activation phenotype (M2) is an anti-inflammatory phenotype that is characterized by the secretion of anti-inflammatory cytokines [51]. Our findings further demonstrated that rhFGF21 attenuated the polarization of microglia toward M1 but have no effect on the M2 phenotype in the acute phase of the MCAO model. Consistently, our in vitro results demonstrated that rhFGF21 hampered the expression of pro-inflammatory cytokines in LPS- or OGD-treated microglia but had no influence on anti-inflammatory genes (IL-10, CD206, and IGF-1). Concomitantly, mice that underwent experimental MCAO exhibited a gradual decrease in the ischemic hemisphere from day 1 to 3 after reperfusion, and the level subsequently returned to preinjury levels by day 7. A similar phenomenon was also described by a previous study [52] in which microglia from the ischemic hemisphere were remarkably reduced at 3 days after stroke. Furthermore, our results revealed the temporal profile of microglial polarization. In accordance with previous research [53], we observed that the levels of the M1-type marker (CD68) significantly increased beginning from day 1 onward. Notably, the expression of the M2 marker CD206 was also increased at 1 day after MCAO, although this increase was no longer observed within 7 days of MCAO injury, which is consistent with the findings of Perego et al. [54]. Taking into consideration the earlier upregulation of IL-10 gene expression, which mediates the shift of microglia to the M2 phenotype, there may be a temporary increase in M2-like microglia at 1 to 3 days post-stroke, and this notion is supported by the results of Kanazawa et al. [4].

Moreover, our study focused on the temporal and spatial presence of migrated immune cells in the acute phase of stroke and highlighted the participation of peripheral macrophages. A previous study [55] showed that the different immune cell types in the post-ischemic area had distinct temporal profiles while the majority of immune cells dramatically accumulated in the ischemic hemisphere at 3 days after stroke. Similarly, in our findings, a massive accumulation of immune cells occurred at 3 days after reperfusion, and the levels were restored back to baseline by day 7. Importantly, rhFGF21 effectively eliminated the invasion of peripheral immune cells. Additionally, rhFGF21 suppressed the activation of peripheral macrophages in the spleen and blood of mice subjected to MCAO, which is consistent with previous reports showing that FGF21 reduced macrophage-mediated inflammation by NF-κB in RWA264.7 macrophages [29]. These findings suggest that the anti-inflammatory effects of rhFGF21 are mediated not only by resident microglia in the brain but also by hematogenous macrophages.

NF-κB is a key transcription factor in the progression of inflammation, and its activation is accompanied by the release of a panel of inflammatory cytokines and chemokines, such as TNF-α, IL-1β, IL-6, and Cox-2 [56, 57]. Indeed, microglia polarization has been proposed to induce multiple mechanisms, including NF-κB signaling pathways [58]. Previous literature demonstrated that the beneficial effects of rhFGF21 on macrophages occur through the inhibition of NF-κB, and our study further validated that rhFGF21 suppresses the activity of NF-κB via FGFR1 in LPS-stimulated murine microglia. In addition, PPAR-γ is a nuclear transcriptional factor [59], and its activation affects not only peripheral systems in ischemia-reperfusion-induced kidney injury and trinitrobenzenesulfonic acid (TNBS)-induced inflammatory bowel disease but also the CNS due to its anti-inflammatory ability [28]. In the present study, rhFGF21 significantly elevated the transcriptional activities of PPAR-γ in LPS-stimulated BV2 cells, which further contributed to anti-inflammation. To recapitulate, rhFGF21, through its actions on FGFR1, suppresses the inflammatory response by modulating the activation of microglia via inhibiting NF-κB and elevating PPAR-γ.

## Conclusions

In summary, our studies demonstrate that the anti-inflammatory effect of rhFGF21 on focal cerebral ischemia occurs through regulation of both central microglia/macrophages and peripheral macrophages via NF-κB and PPAR-γ signaling pathways, indicating that rhFGF21 is a promising candidate for the treatment of ischemic stroke.

## Data Availability

The datasets used and/or analyzed during the current study are available from the corresponding author on reasonable request.

## Abbreviations

MCAO: Transient middle cerebral artery occlusion
BBB: Blood-brain barrier
MACS: Magnetic cell sorting
CCA: Common carotid artery
ECA: External carotid artery
ICA: Internal carotid artery
FACS: Fluorescence-activated cell sorting
tPA: Tissue plasminogen activator
TNF-α: Tumor necrosis factor-α
iNOS: Inducible nitric oxide synthase
IL-1β: Interleukin-1β
IL-6: Interleukin-6
TGF-β: Transforming growth factor beta
IGF-1: Insulin-like growth factor 1
IL-10: Interleukin-1
IL-4: Interleukin-4
MCP-1: Monocyte chemoattractant protein-1
CXCL1: Chemokine C-X-C motif ligand 1
HBSS: Hank’s balanced salt solution
PBS: Phosphate-buffered saline
ANOVA: One-way analysis of variance
CNS: Central nervous system
rhFGF21: Recombinant human fibroblast growth factor 21
NF-κB: Nuclear factor-kappa B
PPAR-γ: Peroxisome proliferator-activated receptor-γ
